# Nonadiabatic Nano-optical Tunneling of Photoelectrons in Plasmonic Near-Fields

**DOI:** 10.1021/acs.nanolett.1c04651

**Published:** 2022-03-04

**Authors:** Béla Lovász, Péter Sándor, Gellért-Zsolt Kiss, Balázs Bánhegyi, Péter Rácz, Zsuzsanna Pápa, Judit Budai, Christine Prietl, Joachim R. Krenn, Péter Dombi

**Affiliations:** † Wigner Research Centre for Physics, 1121 Budapest, Hungary; ‡ ELI-ALPS Research Institute, 6728 Szeged, Hungary; § Institute of Physics, University of Graz, 8010 Graz, Austria

**Keywords:** ultrafast plasmonics, photoemission, strong-field phenomena, nano-optical near-field, femtosecond processes

## Abstract

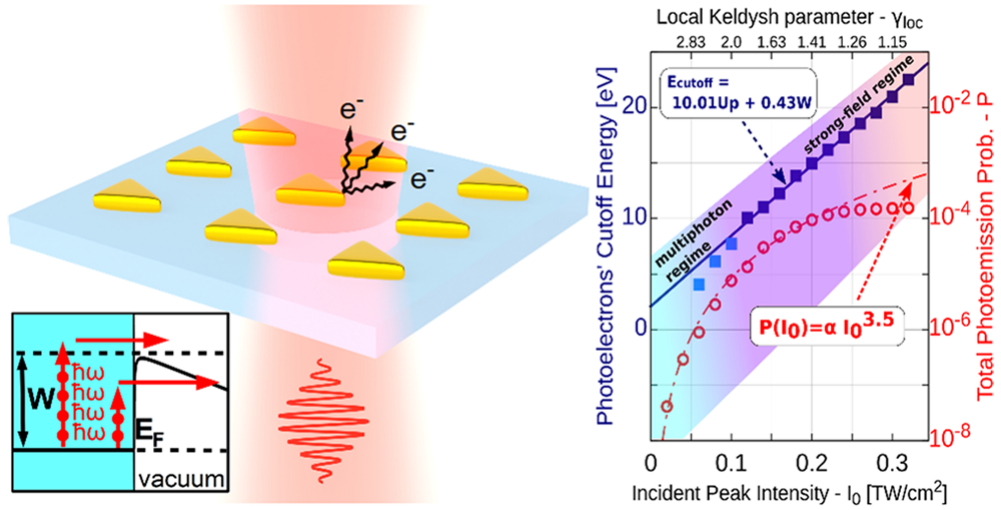

Nonadiabatic nano-optical electron tunneling in the transition region between multiphoton-induced emission and adiabatic tunnel emission is explored in the near-field of plasmonic nanostructures. For Keldysh γ values between ∼1.3 and ∼2.2, measured photoemission spectra show strong-field recollision driven by the nanoscale near-field. At the same time, the photoemission yield shows an intensity scaling with a constant nonlinearity, which is characteristic for multiphoton-induced emission. Our observations in this transition region were well reproduced with the numerical solution of Schrödinger’s equation, mimicking the nanoscale geometry of the field. This way, we determined the boundaries and nature of nonadiabatic tunneling photoemission, building on a key advantage of a nanoplasmonic system, namely, that high-field-driven recollision events and their signature in the photoemission spectrum can be observed more efficiently due to significant nanoplasmonic field enhancement factors.

Even though Keldysh’s original paper^1^ laid the theoretical groundwork for much of today’s investigations of strong-field physics for both atomic and solid-state systems, strong-field phenomena were initially explored mostly in atomic and molecular physics scenarios.^2^ On the other hand, investigation of photoemission features was slower in the condensed matter physics community. Regardless, in either class of systems, the Keldysh scale parameter (γ) provides a guide to the nature of the bound-free transition (photoionization or photoemission) mechanism, especially in the limiting cases. The Keldysh parameter is derived from the ratio of the work function (or ionization potential) and the so-called ponderomotive energy which is the average kinetic energy of a free electron in the field of a certain optical wave. For higher optical frequencies and moderately strong fields, with the Keldysh scale parameter being well above one (γ ≫ 1), multiphoton-induced photoemission dominates, with the photocurrent following the intensity envelope of the laser pulse, raised to the multiphoton order.^3^ Upon decreasing the optical frequency and/or increasing the laser intensity (γ ≪ 1), tunneling of the electrons takes over with the photocurrent being ejected adiabatically with the oscillating field into the continuum.^3^ However, this general understanding of fundamental light–matter interaction processes does not involve any prediction whether there is a γ range where both multiphoton and strong-field features are present or on the exact physical processes taking place in such a transition regime. Here, we show results that provide answers to both questions.

A number of contemporary experiments in atomic and molecular physics^4−7^ and also nano-optics^8,9^ are conducted in the intensity range between these two extremes. However, systematic experimental research highlighting the nature of this transition itself is missing. There are a few works where one can follow some sort of evolution of the electron emission and rescattering process as the Keldysh parameter is tuned, but either the tuning is implicit and hence not transparent,^10^ or it is explicit but is confined to a region where only one emission process dominates.^11,12^ The question naturally arises on what happens in the transition region around γ ∼ 1. In this work, we aim to identify the Keldysh value for the latter transition. Theoretical work on 1D and 2D H-atom-like systems^13,14^ analyzed the γ value for such a transition, determining it to be ∼2. A combined experimental/theoretical work with gold nanotips confirmed this estimate,^15^ contributing to the unified picture of laser–atom and laser–solid interactions. A further study^8^ links the onset of a delayed emission mechanism from a tungsten tip to that of AC tunneling at γ ≈ 2.3. Based on the analysis of a quantum model of a 1D solid interface, a simple formula (), where *W* is the work function of the metal, was proposed for the lower bound of the transition between the multiphoton and tunneling regimes,^16^ resulting in γ = 2.5 for tungsten and 2.7 for gold. In contrast, ref (17) reported on the multiphoton–tunnel transition taking place at γ ≈ 0.9. These works indicate a relatively broad intensity range where we can expect nonadiabatic tunneling features.

Here, we demonstrate photoemission between the regimes of multiphoton-induced photoemission and the nonadiabatic tunneling of electrons at the surface of gold nanoparticles. We answer both the question on the stretch of this transition regime and the physical processes taking place there. To accomplish this, we exploit plasmonic methods enabling nanometer-scale field localization and enhancement^18,19^ as well as near-field probing at the same time.^20−22^ We analyze distinct plateau-like structures that appear in the photoelectron spectra. Even though analogous plateau features have been observed in the high-order above-threshold ionization spectra of atoms;^4^ however, the electron rescattering process is significantly more efficient in the case of solid-state systems, making it significantly easier to detect. This way, we will show that, in the transition region, tunneling/rescattering of electrons takes place at the same time when the electron current depends on the photocurrent in a manner that is characteristic for multiphoton-induced emission.

It is known of photoionization processes in atomic physics that, in the strong-field regime, where tunneling electron emission sets in, photoelectrons are accelerated in a quasi-classical manner in the laser field, and after rescattering from the parent ion, they can acquire a maximum kinetic energy which is roughly 10 times the ponderomotive potential (*U*_p_) of the laser field, i.e., the average kinetic energy of a free electron moved by the field of the laser. After small quantum mechanical corrections, the corresponding simple formula for the measurable maximum electron kinetic energy was given by *E*_cutoff_ = 10*U*_p_ + 0.54*I*_p_ (see refs (23) and (24)), which is generally considered valid for laser–atom interactions with the ionization potential *I*_p_. At this point, it needs to be validated whether a similar simple relationship can be used in the case of photoemission from metals by replacing the *I*_p_ value with the work function, *W*. To achieve this goal, accurate theoretical models should be considered, adequate for obtaining convergent and correct photoemission spectra. Several well-tested or newly developed quasi-classical models (consisting of procedures that consume relatively lower CPU time than the ab initio methods) are widely used to investigate the tunneling process (placing their focus on gaseous targets^25^ or on the field-induced photocurrents inside metallic nanogaps^26^). However, considering that our main interest lies in features in the photoemission spectrum, we considered a pure quantum picture of the system under study. Hence, we constructed a quantum mechanical model and determined the scaling law that can be applied for our case, i.e., photoemission into plasmonic near-fields. Within our model, we solved numerically the 1D time-dependent Schrödinger equation (TDSE):
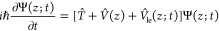
1by employing a mixed split operator and Crank–Nicolson approach,^27^ where for the length gauge form *V*_le_(*z*;*t*) = *zE*_loc_(*z*;*t*) electron–laser interaction term we have also included a *Q* (*z*) field enhancement factor, i.e., *E*_loc_ = *E*_in_(*t*)*Q*(*z*). Here *Q*(*z*) was a decreasing function and was obtained by fitting an exponential curve after taking into account the average field enhancement values at a number of discrete distances *z*_i_ ≥ 0 (i ∈ {1,2,...}) of plasmonic nanorods. The used field enhancement values were acquired beforehand by finite-difference time-domain (FDTD) and electromagnetic simulations.^28^ The incident fields considered for the simulation were Gaussian pulses centered at 800 nm with pulse durations of τ = 5.3 fs at full width at half-maximum (fwhm) intensity. Here, the Ψ_0_ = Ψ(*z*;*t* = 0) initial wave function (WF) of the electron, located on the Fermi level in the bulk and described by the *V*(*z*) = −exp[−β(*z* + |*z*|)] [2(*z* + |*z*|) + 1/(*E*_F_ + *W*)]^−1^ potential, was obtained by diagonalizing the field-free *H*_0_ = *T* + *V* Hamiltonian matrix represented on a finite-difference grid. The parameter β in the local potential represents the screening constant that describes the shielding effect of the bulk electrons on the active electron. Its value was set to be 0.6. This proved to provide an appropriate description of the real physical picture, yielding good agreement with experimental data.^23^

Figure 1a shows the photoelectron spectra (projections of the photoemission wavefunction onto continuum plane wave states) calculated after the passage of the laser pulse for different *I*_0_ peak field intensities. The assumption that in the final time moment the emitted electron can be correctly described using simple plane waves holds since a major part of the wave packet already departed far from the vicinity of the local potential (i.e., from the metallic surface), driven away by the incoming oscillating field. Hence, in the proximity of the relevant photoelectrons, the external electric field strength (a combination of the local potential and the driving laser field) has been practically reduced to zero once the laser pulse has left. As one can observe, a clearly distinguishable plateau feature started to appear from *I*_0_ ≥ 0.12 TW/cm^2^. By introducing the concept of the local Keldysh parameter γ_loc_, which is defined using the maximum of the plasmonically enhanced local field on the nanoparticle’s surface (*z* = 0), i.e., γ_loc_ = ω(2*W*)^1/2^/*E*_loc,max_, with *E*_loc,max_ = *E*_0_*Q* (*z* = 0), one can observe in Figure 1 that the aforementioned intensity values correspond to γ_loc_ ≤ 1.8. For the higher energy part (the plateau and the roll-off region) of these spectra, we fitted a four-parameter model function *f*_mod_(*x*) = *c* – *a*(*x* – *b*)[1 – exp(−*d*(*x* – *b*))]^−1^, which, in the limiting case lim_*x*→*b*_, takes the value *c* – *a*/*d* (dotted line in Figure 1a). We took the value of the *b* parameter, i.e., the intersection point of the two linear lim_ε→∓∞_(*f*_mod_) asymptotes, as the photoelectron cutoff energy: *E*_cutoff_ = *b*. In Figure 1b, we show the obtained cutoff energy values as a function of different peak intensities. By fitting, we determined the scaling:
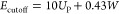
2It is worth noting here that the quantum mechanical correction term was found to be only slightly different from the 0.54*W* value obtained for atomic targets.^24^ This way, we proved the universal applicability of the 10*U*_p_ scaling.

**Figure 1:**
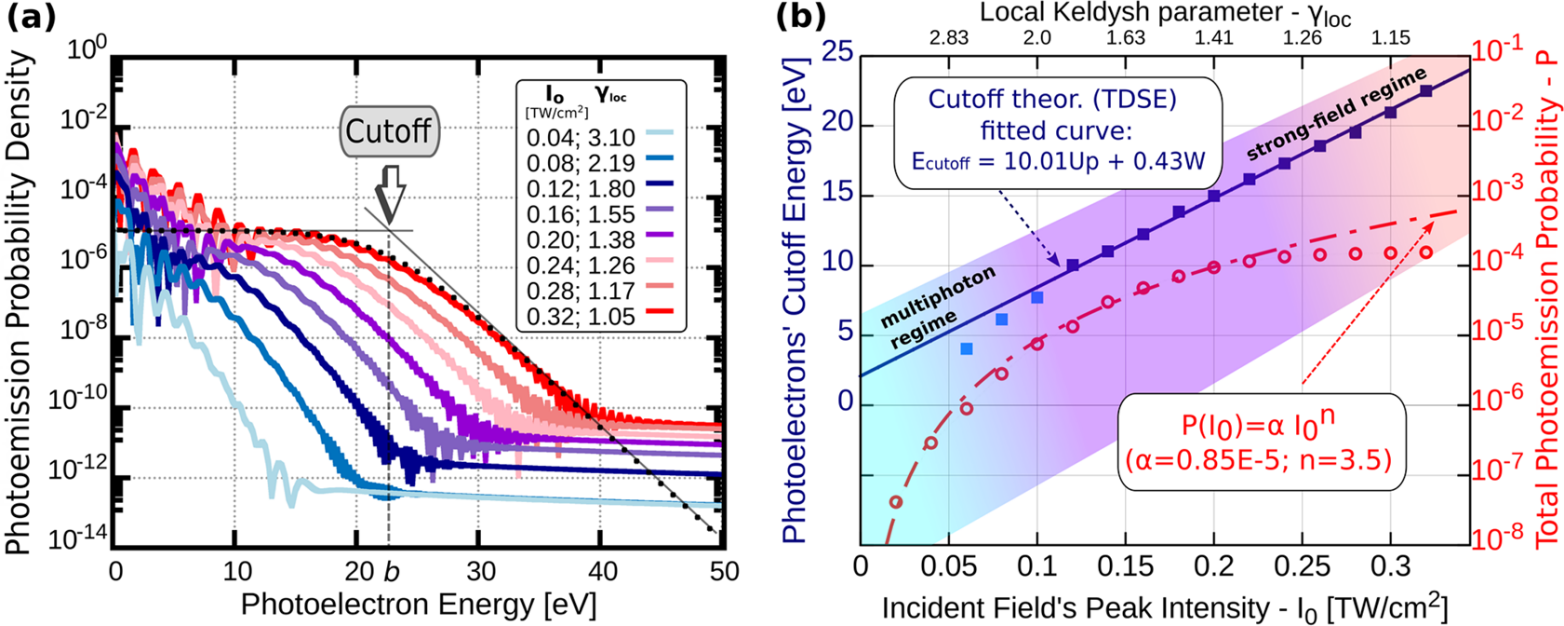
(a) Calculated photoelectron spectra (photoemission probability density) for different *I*_0_ incident peak intensities, 800 nm central wavelength, 5.3 fs pulse length, and 5.3 eV work function (gold). Field enhancement and its decay are taken into account with a *Q*(*z*) exponentially decaying curve. The electron spectral cutoff energy is defined as the parameter *b* of the *f*_mod_ model function for fitting the plateau rolloff region. (b) The upper curve shows the scaling of these cutoff energies (solid blue squares) with the incident peak intensity, *E*_cutoff_ = 10*U*_p_ + 0.43*W*. The onset of rescattering takes place at around γ ∼ 2. The lower curve shows final (*t* = τ) total ionization probabilities (circles) together with the P(*I*_0_) ∼ *I*_0_^3.5^ power function (dashed red line). With the shaded areas, we indicate the multiphoton, transition, and strong-field regimes, respectively. Note that for roughly 1.3 < γ < 2, both a multiphoton-type emission scaling and a strong-field cutoff scaling are present.

In addition, we also calculated the final total ionization probability as a function of *I*_0_ (red circles in Figure 1b), and a transition from the multiphoton to the strong-field regime could be clearly identified starting from the local Keldysh parameter of γ_loc_ ≤ 1.4. Also, by considering that the plateau feature that started to appear in the spectra from γ_loc_ ≤ 2, we showed that the transition between the two regimes starts within the region of γ_loc_ ∈ [1.4, 2], providing a reliable estimate and initial answer to one of our fundamental research questions.

After having a verified simple scaling law at hand (eq 2), we could study how photoemission from different plasmonic nanoparticles takes place in electromagnetic hot spots of our samples. We used laser pulses with octave-spanning bandwidth that were generated by a commercial Ti:sapphire laser oscillator (Venteon Pulse One) at a repetition rate of 80 MHz. The pulses were compressed to ∼7.2 fs duration in case of sample A (see Table 1) and 10.7 fs for samples B and C by a combination of chirped mirrors (Layertec 103366), a pair of fused silica wedges, and plane-parallel fused silica slabs. Characterization of the pulse duration was performed using interferometric FROG (IFROG) and d-scan techniques (see inset of Figure 2a). The laser pulse energy was controlled by a neutral density filter.

**Figure 2:**
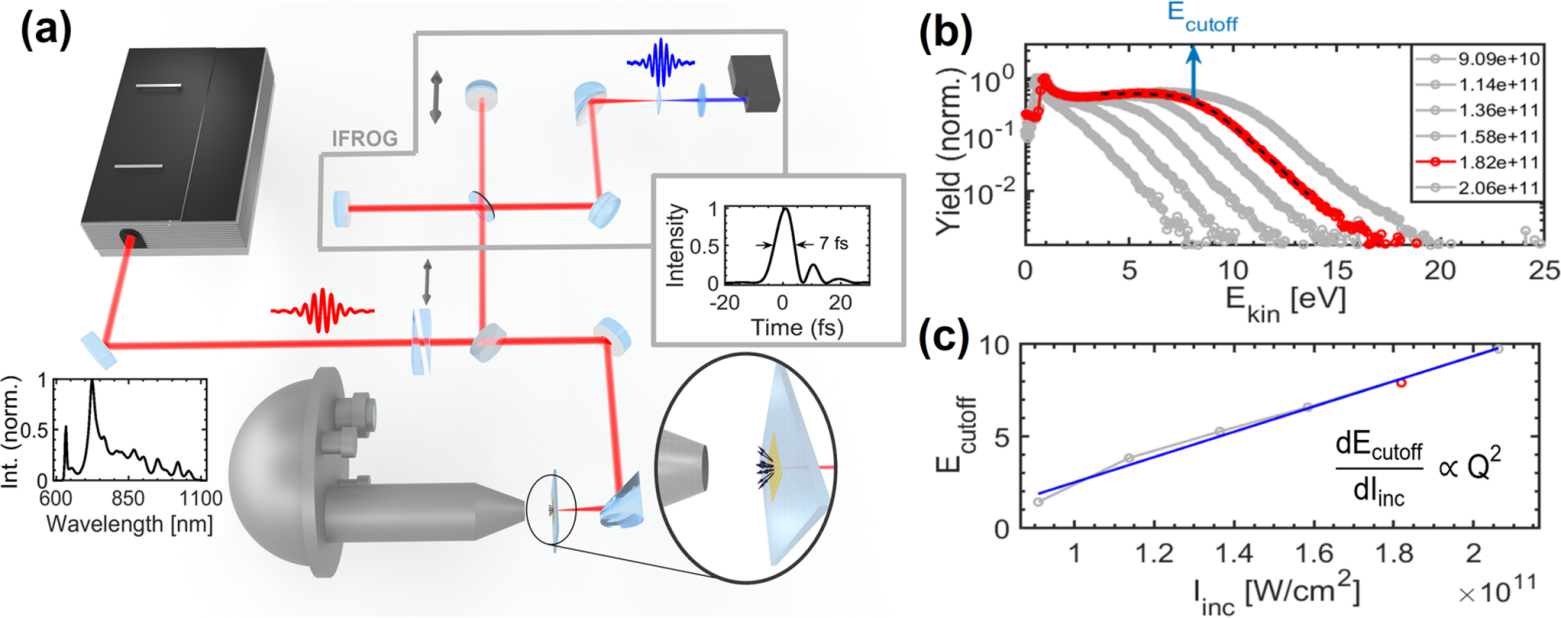
(a) Scheme of the experimental setup. Few-cycle laser pulses from a Ti:sapphire oscillator are focused by an off-axis parabolic mirror and illuminate the sample in transmission. Photoemitted electrons are detected by a hemispherical energy analyzer. Insets: Measured laser spectrum and reconstructed temporal pulse profile in case of sample A. (b) Typical measured photoemission spectra for different laser intensities (the values are given in the legend in units of W/cm^2^). (c) Cutoff energies extracted from the spectra.

**Table 1:** Summary of the Measured and Calculated Field Enhancement Values (See Supporting Information for Details)

		field enhancement	
nanostructure		measured	calculated
A	triangle (160 nm × 100 nm), Figure 3a	9.4 ± 0.6	8.3 ± 2.2
B	rod (192 nm × 103 nm), Figure 3b	12.2 ± 0.3	10.7 ± 1.8
C	triangle (50 nm × 200 nm), Figure 3c	15.0 ± 0.6	13.8 ± 1.3

The sample was housed in a high-vacuum chamber (base pressure: <10^–7^ mbar) and was positioned with nanometer accuracy in all three dimensions using stacked piezostages (Attocube ECS 3030). Laser pulses with linear polarization were focused to a spot of ∼7.4 μm in diameter (1/*e*^2^) with an off-axis parabolic mirror and illuminated the sample from the back side. Electrons emitted from the surface of the nanostructures entered a hemispherical energy analyzer (SPECS Phoibos 100 R7). Data acquisition was controlled using the SpecsLab Prodigy software by setting an energy width of 0.1 eV and scanning center of the energy window between 0.4 eV and typically 50 eV. The spectra shown on the top right inset of Figure 2 were collected with an entry slit width of 3 mm and an open exit slit. To avoid detector saturation, the signal was reduced by a factor of 20–30 using a laser beam chopper to let only a small portion of the pulses through to the experiment. The Earth’s ambient magnetic field is compensated with three sets of Helmholtz coils, seated outside of the vacuum chamber.

For a given nanostructure, a series of spectra were collected at different incident laser intensities. At low laser intensities, the spectra show a narrow low-energy peak and an exponential “tail” at higher electron kinetic energies (a straight line on a semilogarithmic graph; see Figure 2b). These correspond to direct electrons (i.e., those that are accelerated but not rescattered in the local fields). As the laser intensity is increased, the spectra develop a distinct plateau-like feature at higher electron kinetic energies, in accordance with our simulation results. This signals the appearance of electrons that are ejected, accelerated, and rescattered in the time-varying enhanced local fields. We analyze this portion of the electron energy distributions to determine the strength of the local fields with the method presented in refs (21) and (22). At each laser intensity, the electron cutoff kinetic energy was determined from the associated spectrum. We substitute the expression for the ponderomotive energy (*U*_*p*_) to eq 2 to find that the cutoff energy is a linear function of the peak intensity, and its slope is proportional to the square of the nanoplasmonic field enhancement.
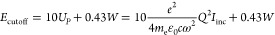
3Here, ω is the central angular frequency of the laser, *W* is the work function of the metal (∼5.3 eV for gold in our case), *E*_inc_ is the electric field of the incident laser radiation, *Q* is the local field enhancement (*E*_loc_ = *QE*_inc_), *m*_e_ is the electron mass, and *e* is the elementary charge. We use this relationship to determine the field enhancement by linearly fitting the cutoff energy versus peak intensity curve (see Figure 2b,c for illustration and the Supporting Information for details of this procedure).

Figure 3 shows the overview of the results. In panels (a–c), we show SEM images of nanostructures A, B, and C used in the experiment (for designations, see Table 1; the incident laser field is polarized horizontally, along the scale bar). In each column of Figure 3, the data corresponding to each nanostructure are present. The photoelectron yield versus incident laser intensity (bottom axis) is plotted on a double logarithmic scale in panels (j–l). In the same panels, the local Keldysh parameter (γ_loc_) is indicated (top axis). Linear fit (red solid line) to the raw data (black circles) shows slopes between 2.9 and 3.6, indicative of photoelectron emission due to absorption of 3–4 photons from the incident laser field. These are reasonable numbers given that our laser spectrum spans from 630 to 1100 nm with a central wavelength of 803 nm (corresponding to 1.54 eV photons) and the nominal value of 5.3 eV for the work function. These are also in accordance with results of our simulations (see Figure 1b).

**Figure 3:**
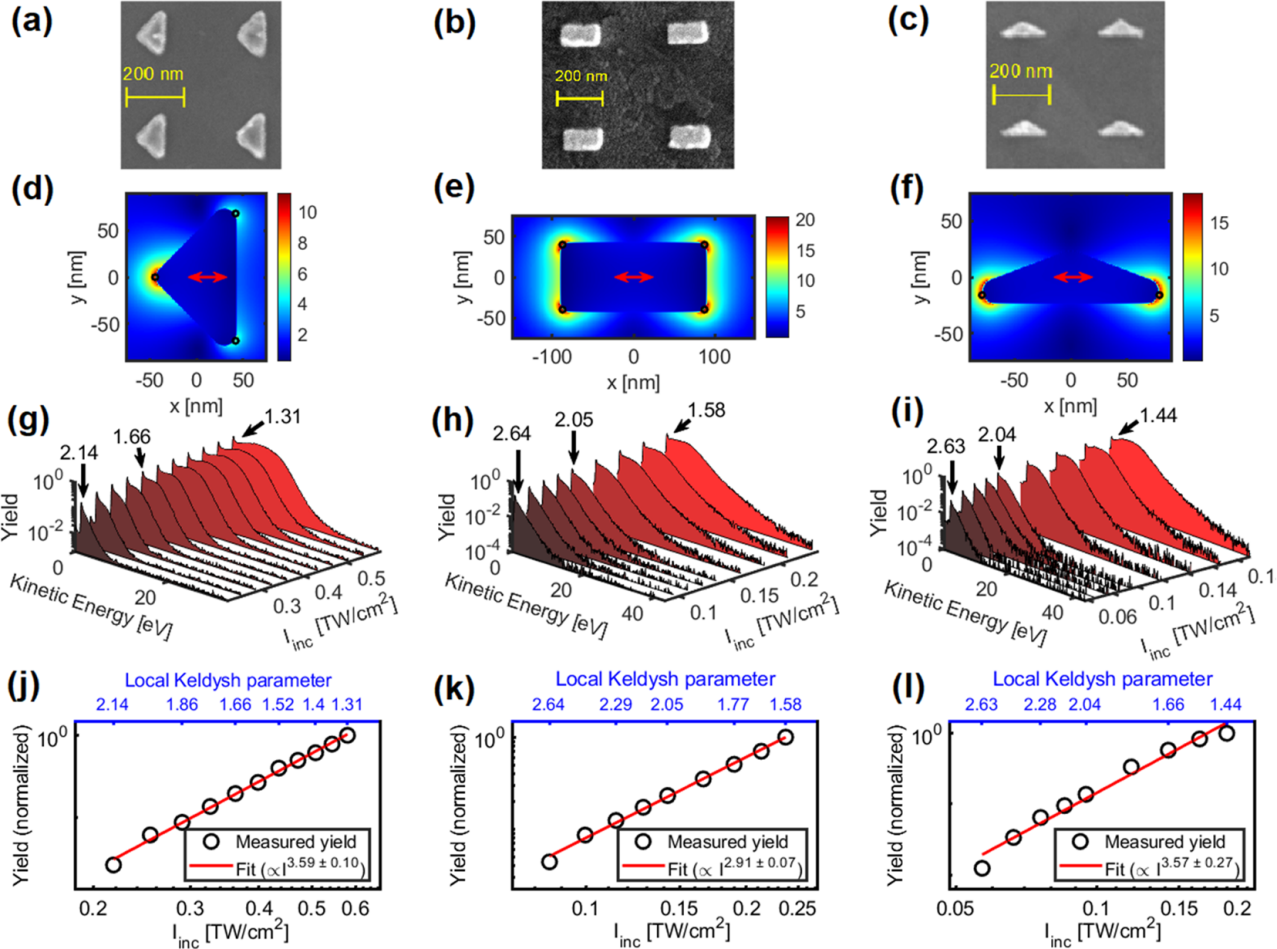
(a–c) SEM images of the gold nanostructures. The laser polarization was linear and horizontal along the scale bar in all cases. (d–f) Spatial distributions of the field enhancement values from FDTD simulations in a plane at a height of ∼15 nm above the substrate surface. Black circles mark monitor points in these planes; these and further monitor points in other planes were used for calculating the average field enhancement values in Table 1. (g–i) Measured photoemission spectra for different incident laser intensities, with the local Keldysh parameter values indicated with arrows for some of the spectra. (The yield is normalized to the global maximum value of each data set and shown on a logarithmic scale.) (j–l) Photoemission yields as a function of incident peak laser intensity (black circles) and linear fits (solid red lines). The nonlinear exponents are ∼3.6 for the triangular nanostructures and ∼2.9 for the nanorods.

The shapes of the photoemission spectra for increasing laser intensities are shown in Figure 3g–i. For the lowest incident intensities, spectra consist of a low-energy peak and an exponential falloff (straight line on the semilogarithmic plot, hence the spectra are “triangle-shaped”). Such spectra are well-known to be observed under conditions γ_loc_ ≫ 1, i.e., in the multiphoton regime.^29^

Upon increasing the laser intensity, a plateau feature gradually appears, giving evidence of the appearance of rescattering electrons with substantially increased kinetic energy. The plateau/cutoff feature enables the determination of the field enhancement (see Table 1 and refs (21) and (22), along with the magnitude of the local field and the local Keldysh parameter. The fact that there is excellent agreement between measured and calculated field enhancement values (Table 1) confirms the existence of rescattering electrons. Earlier work^17^ also suggests that in the case the quiver amplitude of the electrons is much smaller than the local field decay length (i.e., the adiabacity parameter δ > 1), then the appearance of the plateau electrons signifies that the Keldysh parameter is γ_loc_ < 1; that is, tunneling starts to play an important role in the ionization process.

The appearance of the plateau is observed at Keldysh parameter values of about 1.99, 2.29, and 2.28. This is in very good agreement with our theoretical results in Figure 1, showing the appearance of 10*U*_p_ electrons for γ_loc_ < 2.2, representing the onset of strong-field effects. However, at the same time, for 1.3 < γ_loc_ < 2.2 in the nanoplasmonic near-field, the power-law scaling of the photocurrent with laser intensity also holds true with a constant exponent. Thus, strong-field electron acceleration features and multiphoton scaling laws are present at the same time in this transition region, which can be termed as the nonadiabatic tunneling regime. This is in accord with experiments highlighted in ref (8) but not with the calculations shown in ref (16). In the latter, a hallmark of the onset of tunnel emission is the deviation from the smooth power-law dependence. Thus, our work highlights the importance of considering the spectral signatures of the rescattered electrons, in addition to the dependence of the emitted yield on peak intensity.

In summary, we demonstrated nonadiabatic tunneling photoemission in few-cycle near-fields in the vicinity of various plasmonic nanostructures. By doing so, we pointed out the regime where multiphoton emission scaling laws and strong-field electrons are present at the same time. By analyzing these electron spectra and determining plasmonic field enhancement with spectral cutoffs, we could show the presence of ponderomotively accelerated electrons in this nonadiabatic region, which is perfectly characterized by multiphoton emission scaling laws. Nonadiabatic tunneling photoemission takes place for Keldysh γ values between ∼1.3 and ∼2.2. With these experiments and the corresponding support theory, we answered both the question of (i) which is the characteristic intensity region where both multiphoton and strong-field emission features are present and (ii) what are the typical emission mechanisms in this transition region. For the latter, we showed that measurable rescattering of the electrons can take place even when the emission is perfectly characterized by multiphoton scaling laws.

## Supporting Information Available

Determination method of the cutoff energies and field enhancement values; control measurements; nanostructure damage examination; details of the finite-difference time-domain simulations; and few-cycle pulse characterization (PDF)

## References

[ref1] Fedorov M. V. L.V. Keldysh's Ionization in the Field of a Strong Electromagnetic Wave. Sov. Phys. JETP 2016, 122 (3), 449–455. 10.1134/S1063776116030043.

[ref2] Muller H. G.; Agostini P.; Petite G.; Freeman R. R.; Bucksbaum P. H.; Cooke W. E.; Gibson G.; Mcilrath T. J.; van Woerkom L. D.; Gallagher T. F.; Corkum P. B.; Burnett N. H.; Brunel F.; L’Huillier A.; Lompre L.-A.; Mainfray G.; Manus C.; Luk T. S.; McPherson A.; Boyer K.; Rhodes C. K.; Kulander K. C.; Schafer K. J.; Krause J. L.; Eberly J. H.; Grobe R.; Law C. K.; Su Q.; Lambropoulos P.; Tang X.; Potvliege R. M.; Shakeshaft R.; Gavrila M. In Atoms in Intense Laser Fields; Gavrila M., Ed.; Cambridge University Press: Cambridge, UK, 1992.

[ref3] Yudin G. L.; Ivanov M. Y. Nonadiabatic Tunnel Ionization: Looking inside a Laser Cycle. Phys. Rev. A - At. Mol. Opt. Phys. 2001, 64 (1), 4 10.1103/PhysRevA.64.013409.

[ref4] Becker W.; Goreslavski S. P.; Milošević D. B.; Paulus G. G. The Plateau in Above-Threshold Ionization: The Keystone of Rescattering Physics. Journal of Physics B: Atomic, Molecular and Optical Physics. 2018, 162002 10.1088/1361-6455/aad150.

[ref5] Meckel M.; Comtois D.; Zeidler D.; Staudte A.; Pavičić D.; Bandulet H. C.; Pépin H.; Kieffer J. C.; Dörner R.; Villeneuve D. M.; Corkum P. B. Laser-Induced Electron Tunneling and Diffraction. Science 2008, 320 (5882), 1478–1482. 10.1126/science.1157980. 18556555

[ref6] Werby N.; Natan A.; Forbes R.; Bucksbaum P. H. Disentangling the Subcycle Electron Momentum Spectrum in Strong-Field Ionization. Phys. Rev. Res. 2021, 3, 23065 10.1103/PhysRevResearch.3.023065.

[ref7] Sándor P.; Sissay A.; Mauger F.; Gordon M. W.; Gorman T. T.; Scarborough T. D.; Gaarde M. B.; Lopata K.; Schafer K. J.; Jones R. R. Angle-Dependent Strong-Field Ionization of Halomethanes. J. Chem. Phys. 2019, 151 (19), 194308 10.1063/1.5121711. 31757133

[ref8] Yanagisawa H.; Schnepp S.; Hafner C.; Hengsberger M.; Kim D. E.; Kling M. F.; Landsman A.; Gallmann L.; Osterwalder J. Delayed Electron Emission in Strong-Field Driven Tunnelling from a Metallic Nanotip in the Multi-Electron Regime. Sci. Rep. 2016, 6 (1), 35877 10.1038/srep35877. 27786287 PMC5082369

[ref9] Shi L.; Babushkin I.; Husakou A.; Melchert O.; Frank B.; Yi J.; Wetzel G.; Demircan A.; Lienau C.; Giessen H.; Ivanov M.; Morgner U.; Kovacev M. Femtosecond Field-Driven On-Chip Unidirectional Electronic Currents in Nonadiabatic Tunneling Regime. Laser Photonics Rev. 2021, 15 (8), 2000475 10.1002/lpor.202000475.

[ref10] Krüger M.; Schenk M.; Hommelhoff P.; Wachter G.; Lemell C.; Burgdörfer J. Interaction of Ultrashort Laser Pulses with Metal Nanotips: A Model System for Strong-Field Phenomena. New J. Phys. 2012, 14 (8), 085019 10.1088/1367-2630/14/8/085019.

[ref11] Herink G.; Solli D. R.; Gulde M.; Ropers C. Field-Driven Photoemission from Nanostructures Quenches the Quiver Motion. Nature 2012, 483 (7388), 190–193. 10.1038/nature10878. 22398557

[ref12] Colosimo P.; Doumy G.; Blaga C. I.; Wheeler J.; Hauri C.; Catoire F.; Tate J.; Chirla R.; March A. M.; Paulus G. G.; Muller H. G.; Agostini P.; Dimauro L. F. Scaling Strong-Field Interactions towards the Classical Limit. Nat. Phys. 2008, 4, 386–389. 10.1038/nphys914.

[ref13] Wang R.; Zhang Q.; Li D.; Xu S.; Cao P.; Zhou Y.; Cao W.; Lu P. Identification of Tunneling and Multiphoton Ionization in Intermediate Keldysh Parameter Regime. Opt. Express 2019, 27 (5), 6471 10.1364/OE.27.006471. 30876249

[ref14] Hao X.; Shu Z.; Li W.; Hu S.; Chen J. Quantitative Identification of Different Strong-Field Ionization Channels in the Transition Regime. Opt. Express 2016, 24 (22), 25250 10.1364/OE.24.025250. 27828463

[ref15] Bormann R.; Gulde M.; Weismann A.; Yalunin S. V.; Ropers C. Tip-Enhanced Strong-Field Photoemission. Phys. Rev. Lett. 2010, 105 (14), 147601 10.1103/PhysRevLett.105.147601. 21230866

[ref16] Pant M.; Ang L. K. Ultrafast Laser-Induced Electron Emission from Multiphoton to Optical Tunneling. Phys. Rev. B 2012, 86, 45423 10.1103/PhysRevB.86.045423.

[ref17] Piglosiewicz B.; Schmidt S.; Park D. J.; Vogelsang J.; Groß P.; Manzoni C.; Farinello P.; Cerullo G.; Lienau C. Carrier-Envelope Phase Effects on the Strong-Field Photoemission of Electrons from Metallic Nanostructures. Nat. Photonics 2014, 8 (1), 37–42. 10.1038/nphoton.2013.288.

[ref18] Novotny L.; Hecht B. Principles of Nano-Optics; Cambridge University Press, 2006.

[ref19] Maier S. A. Plasmonics: Fundamentals and Applications; Springer US, 2007.

[ref20] Dombi P.; Hörl A.; Rácz P.; Márton I.; Trügler A.; Krenn J. R.; Hohenester U. Ultrafast Strong-Field Photoemission from Plasmonic Nanoparticles. Nano Lett. 2013, 13 (2), 674–678. 10.1021/nl304365e. 23339740 PMC3573732

[ref21] Rácz P.; Pápa Z.; Márton I.; Budai J.; Wróbel P.; Stefaniuk T.; Prietl C.; Krenn J. R.; Dombi P. Measurement of Nanoplasmonic Field Enhancement with Ultrafast Photoemission. Nano Lett. 2017, 17 (2), 1181–1186. 10.1021/acs.nanolett.6b04893. 28094992

[ref22] Budai J.; Pápa Z.; Márton I.; Wróbel P.; Stefaniuk T.; Márton Z.; Rácz P.; Dombi P. Plasmon-Plasmon Coupling Probed by Ultrafast, Strong-Field Photoemission with < 7 Å Sensitivity. Nanoscale 2018, 10 (34), 16261–16267. 10.1039/C8NR04242J. 30124717

[ref23] Yalunin S. V.; Herink G.; Solli D. R.; Krüger M.; Hommelhoff P.; Diehn M.; Munk A.; Ropers C. Field Localization and Rescattering in Tip-Enhanced Photoemission. Ann. Phys. 2013, 525 (1–2), L12–L18. 10.1002/andp.201200224.

[ref24] Busuladžić M.; Gazibegović-Busuladžić A.; Milošević D. B. High-Order above-Threshold Ionization in a Laser Field: Influence of the Ionization Potential on the High-Energy Cutoff. Laser Phys. 2006, 16 (2), 289–293. 10.1134/S1054660X06020149.

[ref25] Lewenstein M.; Balcou Ph.; Ivanov M. Yu.; L’Huillier A.; Corkum P. B. Theory of High-Harmonic Generation by Low-Frequency Laser Fields. Phys. Rev. A 1994, 49 (3), 2117–2132. 10.1103/PhysRevA.49.2117. 9910464

[ref26] Kim S.; Schmude T.; Burkard G.; Moskalenko A. S. Quasiclassical Theory of Non-Adiabatic Tunneling in Nanocontacts Induced by Phase-Controlled Ultrashort Light Pulses. New J. Phys. 2021, 23, 083006 10.1088/1367-2630/ac1552.

[ref27] Crank J.; Nicolson P. A Practical Method for Numerical Evaluation of Solutions of Partial Differential Equations of the Heat-Conduction Type. Math. Proc. Cambridge Philos. Soc. 1947, 43 (1), 50–67. 10.1017/S0305004100023197.

[ref28] Accurately simulate photonic components and circuits: Lumerical; https://www.lumerical.com/products/ (accessed on February 23, 2022).

[ref29] Lang P.; Song X.; Ji B.; Tao H.; Dou Y.; Gao X.; Hao Z.; Lin J. Spatial- and Energy-Resolved Photoemission Electron from Plasmonic Nanoparticles in Multiphoton Regime. Opt. Express 2019, 27 (5), 6878 10.1364/OE.27.006878. 30876264

