# Supplementary material for: Nonadiabatic Nano-optical Tunneling of Photoelectrons in Plasmonic Near-Fields
Source: Nano Lett. 2022 Mar 4;22(6):2303–8. doi: 10.1021/acs.nanolett.1c04651 (PMC8949759; doi:10.1021/acs.nanolett.1c04651)
Supplement: Supplementary file 1 — nl1c04651_si_001.pdf [file nl1c04651_si_001.pdf]

Nonadiabatic nanooptical tunneling of photoelectrons in plasmonic near-fields Supporting Information

Béla Lovász╨◊*, Péter Sándor*╨◊*, Gellért-Zsolt Kiss*╨*, Balázs Bánhegyi*╨*, Péter Rácz*╨*,*

Zsuzsanna Pápa╨,╫*, Judit Budai*╫*, Christine Prietl*╟*, Joachim R. Krenn*╟*, Péter Dombi*╨,╫,*

╨Wigner Research Centre for Physics, 1121 Budapest, Hungary
╫ELI-ALPS Research Institute, 6728 Szeged, Hungary
╟Institute of Physics, University of Graz, 8010 Graz, Austria
◊These authors contributed equally to this work
*Corresponding author: dombi.peter@wigner.hu

S.1 Determination of the cutoff energies and field enhancement values

For a given incident laser peak intensity, the cutoff energy was determined by fitting a function that well describes the plateau part of the corresponding photoemission spectrum (Fig. S1.(a)). Our choice for this model function was:

$$log_{10}(Y) = A_0 - A_1 \frac{E_{kin} - A_2}{1 - e^{-A_3(E_{kin} - A_2)}}$$

where E_{kin} is the measured electron kinetic energy, Y is the photoemission yield, and A_n are fit parameters; the estimates of A_0 and A_1 describe the signal level of the plateau and the rapidness of the fall-off of the high-energy tail of the spectrum, respectively; A_2 gives the value of E_{cutoff}; A_3 is related to the radius of curvature of the transition from the flat plateau region to the fall-off.

A single field enhancement value is obtained by fitting a straight line to the appropriate portion of the cutoff energy vs. incident peak laser intensity graph (Fig. S1.(b)). The portion which is included in the fit is selected by visually inspecting the corresponding spectra and confirming that a significant plateau feature is present. Keldysh parameter values are calculated from the local field strengths, i.e., taking into account the obtained field enhancement.

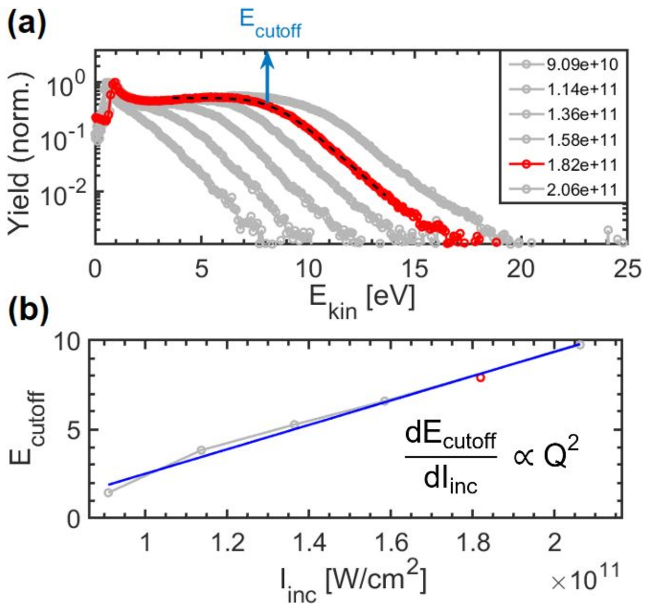

Figure S1. (a) Typical measured photoemission spectra. The legend shows the incident peak laser intensities in W/cm². Black dashed line shows the fit from which the value of

E_{cutoff} can be extracted (see text). **(b)** Cutoff energies (extracted from the spectra) vs. incident laser intensities (black and red circles) and linear fit (solid blue line).

S.2 Control measurements on off-resonant nanostructures and a 20 nm thick gold layer

To further underline the validity of our experimental findings, we have done control measurements on 2 kinds of off-resonant nanostructures, one with triangular base (280 nm × 280 nm; base × altitude), and one with rectangular base (320nm × 130nm), in both cases the first dimension was parallel to the lasers polarization. The height of the nanostructures is 40 nm. Furthermore, a sample with a 20 nm gold layer was also investigated. In the case of the off-resonant nanostructures, the measurements yielded 1 order of magnitude less photocurrent, in comparision with results on resonant samples. Measured spectra have an exponentially decaying peak at low kinetic energies on a linear scale (a straight line on a semilogarithmic plot), and no plateau structure for even the highest intensities, Fig. S2 red and magenta curves. For the gold layer, 2 orders of magnitude lower photocurrent could be measured compared to the off-resonant nanostructures, with similarly narrow spectra, see Fig. S2 blue curve. We would like to point out that since there is no plateau structure in the above mentioned cases even at the highest illuminating intensities, the value of the field enhancement cannot be determined using our method.

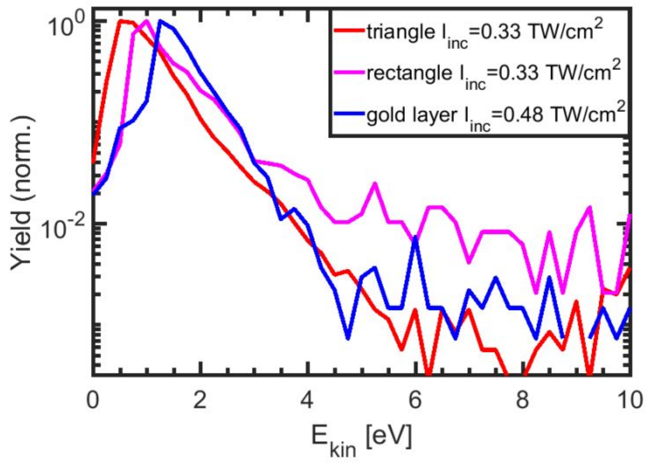

Figure S2. Control measurements on 2 kinds of off-resonant nanostructures and a 20 nm thick gold layer. Note that for the measurements on the nanostructure with the rectangle base (pink curve) the signal-to noise is poorer and so the instrumental background level is already reached at ≈1e-2 on the normalized plot.

S.3 Nanostructure damage examination and exctinction spectra of the samples

For the highest incident laser intensities damage to the nanostructures was a concern, but typical signs of larger damage, like abrupt change of the spectrum and/or loss of substantial electron signal during data acquisition, was not observed. Our experimental setup makes it possible to find the approximate position where the measurements were conducted on the nanostructure array. To make sure that the nanostructures weren't altered during illumination, we made SEM images from those areas (Fig. S3. (a-c)). On the SEM images for the samples a,b and c, no sign of damage can be detected. Extinction spectra for the samples a,b and c were also measured, they are plotted with the lasers spectrum on Fig S3. (d).

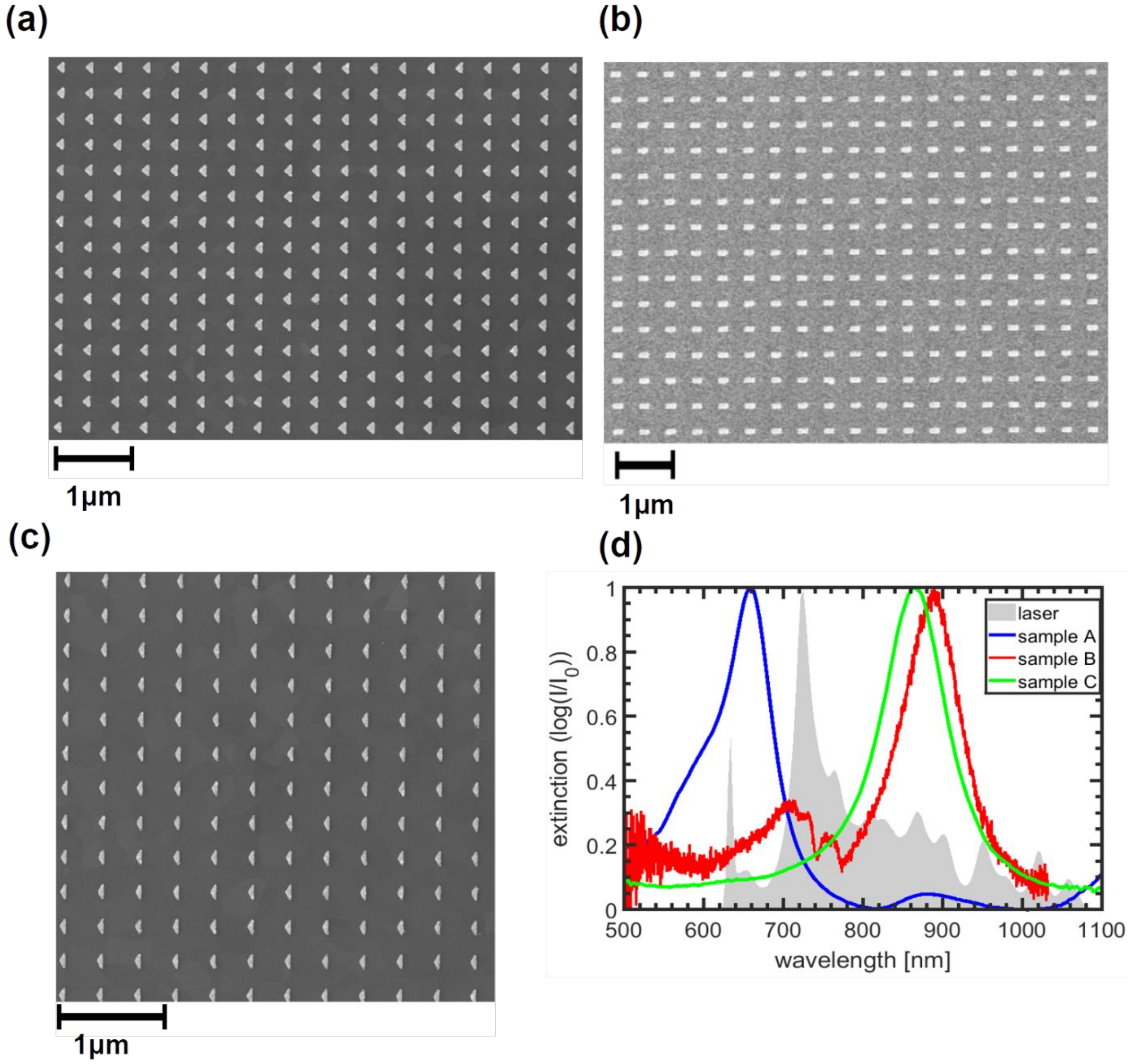

Figure S3. (a-c) SEM images of samples a,b and c approximately at the place of the measurement. **(d)** Extinction spectra for the samples a, b and c.

S.4 Details of the finite-difference time-domain simulations

For modeling the near-field distribution and field enhancement property of the investigated samples, we performed finite-difference time-domain (FDTD) simulations with a commercially available software package (Lumerical FDTD Solutions). The simulated 3D unit cell (350 nm × 350 nm × 1000 nm for the nanotriangles and 500 nm × 500 nm × 1000 nm for the nanorod) contained a gold nanostructure on top of a bulk fused silica substrate covered with a 40 nm thick ITO layer. The substrate was modeled with a constant refractive index n = 1.45. The thickness and optical properties of the ITO were determined by spectroscopic ellipsometric measurement. Optical data of gold was taken from literature[1]. To ensure an accurate description of the nanoobjects' near-field, meshing with a unit size of 0.7 × 0.7 × 0.7 nm^3 was used. Periodic boundary conditions were applied in the directions parallel with the substrate surface, and absorbing boundaries in the perpendicular direction. For excitation, we applied the transform-limited time signal calculated from the spectrum of the laser oscillator used during the measurements. For the calculations, first we included the spectral amplitude and phase of the exciting laser pulse as characterized by the different methods detailed in the SI. Using this data, the full temporal signal of the excitation can be deduced, the amplitude of which was set to unity. The plasmonic nanoparticle has its own spectral amplitude and phase response functions, which can be calculated using FDTD. By applying these response functions to the spectral amplitude and phase of the incoming pulse, we can get the temporal shape of the plasmonic transients. This is exactly what a time monitor in the FDTD does. From the time monitor data, the maximal amplitude of this plasmonic transient at the given spatial points can be extracted. These values are equal with the local field enhancement factor, since the amplitude of the incoming pulse is unity. These field enhancement values were recorded at several points near the nanostructures' corners and the maximal field amplitudes were averaged. With this process, the calculated field enhancement values reflect the full spectral information of the incoming pulse. Since the nanoobject arrays cannot be characterized by a single radius of curvature (R) value, but rather by a radius distribution, the effect of the variation of R on the calculated field enhancement values was investigated. The distribution of R values (see figure S4) was determined using SEM images. Field enhancement calculations were performed at different values of R and the results presented in the manuscript belong to the calculations with R values of the histograms' maximum. The FDTD simulation parameters given above were set according to a strict convergence check.

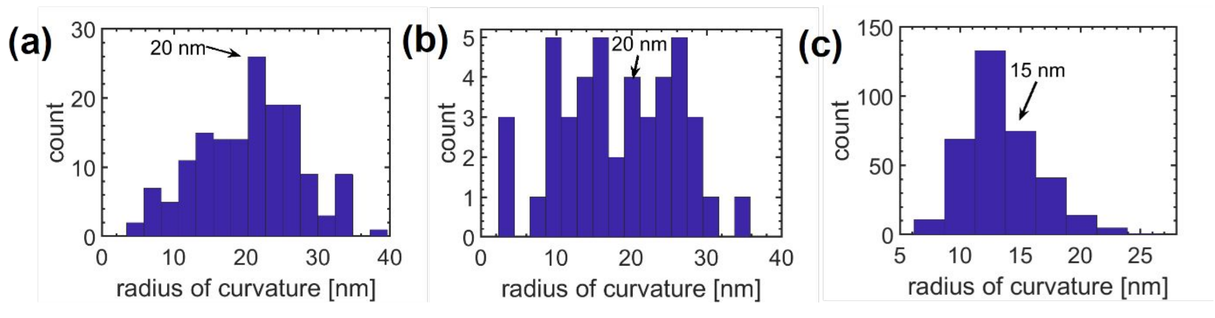

Figure S4. Distributions of corner radii of curvature, extracted from SEM images of the different nanostructures. **(a)** Sample A (160 nm x 100 nm triangles); curvature of 20 nm corresponds to a field enhancement of 8.28. **(b)** Sample B (192 nm x 103 nm rods); curvature of 20 nm corresponds to a field enhancement of 9.96. **(c)** Sample C (50 nm x 200 nm triangles); curvature of 15 nm corresponds to a field enhancement of 12.1.

To check the possible effect of the high intensity excitation on the dielectric function, and more importantly, on the available field enhancement factors, we performed the following analysis. i) We estimated the temperature of the electron subsystem after the arrival of the exciting short pulse with coupled differential equations according to [2]. ii) We took the dielectric function dataset belonging to the observed temperature changes from [3]. iii) We recalculated the field enhancement data with FDTD using the modified optical properties.

The temperature of the electron system considering the average applied laser intensities for each sample was calculated to be Te= 5000 K, 3000 K and 2000 K for sample A, B and C, respectively (Fig. S5 a)). The corresponding dielectric functions along with the room temperature data is plotted in Fig. S3 b). The dataset for the 2000K electron temperature was interpolated using the literature datasets.

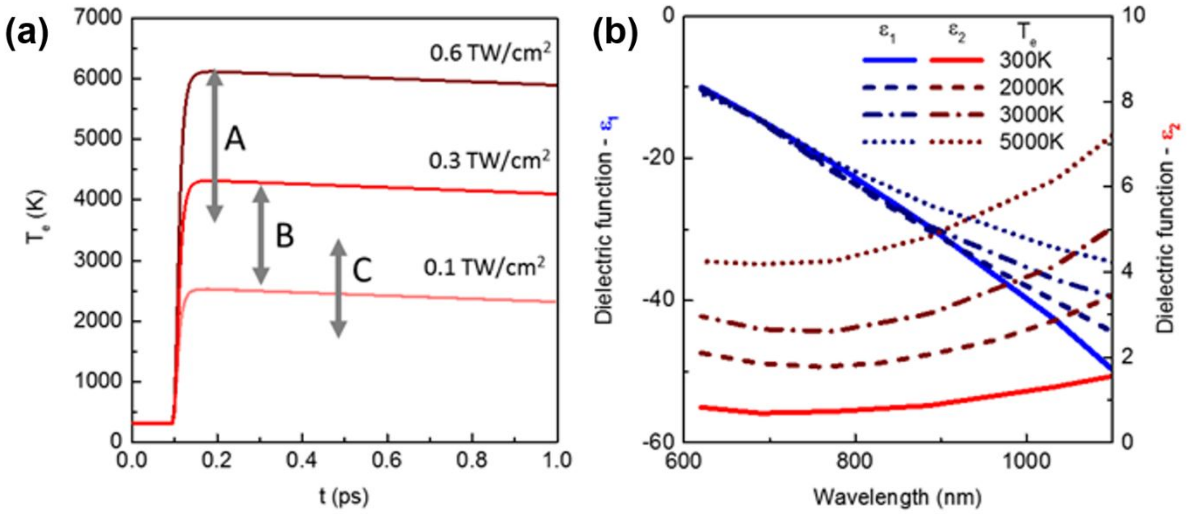

Figure S5. (a) Temperature of the electron system in the first 1ps after the excitation considering different laser intensities. Grey arrows show the applied intensity ranges for the different samples. **(b)** The corresponding curves for the dielectric function (ε_1 and ε_2) of gold at different electron temperatures.

FDTD calculations with the modified optical properties are shown in Table S1. The obtained values are within the error limit for all the samples.

Nanostructure	Calculated field enhancement	
	with room temperature dielectric function	with modified dielectric function
A Triangle (160 nm x 100 nm)	8.3 ± 2.2	7.6
B Rod (192 nm x 103 nm)	10.7 ± 1.8	10.1
C Triangle (50 nm x 200 nm)	13.8 ± 1.3	12.6

Table S1. Comparison of field enhancement values calculated with room temperature or with modified dielectric function.

S.5 Few-cycle pulse characterization

A) IFROG

Duration and temporal profile of the illuminating pulses was determined by Interferometric Frequency-Resolved Optical Gating (IFROG) technique. Briefly, the pulses to be characterized are sent into an interferometer, in which they are split and a variable delay is introduced between them by a closed-loop piezostage (Piezosystem Jena PX200SG). At the output port of the interferometer, the two collinear beams are focused by an f = 25 mm off-axis parabolic mirror into a 10 um BBO crystal. Since the fundamental and the second harmonic beams are orthogonally polarized, the intensity of the former can be almost completely filtered out by a broadband wiregrid polarizer. In practice, we find that in some cases an additional color filter (Schott BG37 or BG39) is needed. The second harmonic signal is detected by a low-noise spectrometer (OceanInsight QEPro).

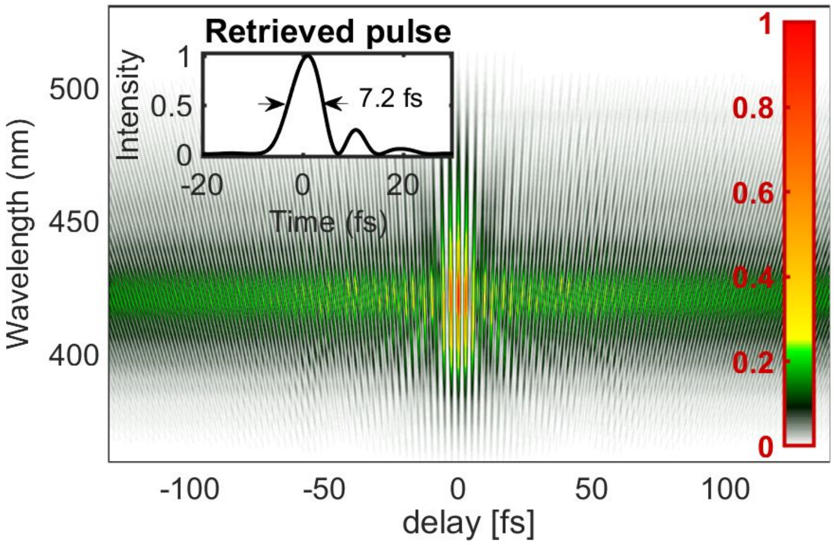

Figure S6. Typical IFROG trace. Inset: reconstructed temporal profile.

Retrieval of the spectral amplitude and phase was done by two separate methods. One is detailed in[4]; while the other one uses the independently measured spectral intensity profile and guesses the spectral phase, in this case taking the form of a Taylor expansion: $\phi(\omega) = \sum_{k=2}^{4} a_k(\omega - \omega_0)^k$. Here, the coefficients a_k and the central frequency ω_0 are adjusted by a simplex search method for the best fit. The two methods both capture significant post-pulse structure after the main pulse (due to uncompensated 3rd and higher-order chirp), and report FWHM-s for the main pulse that are different by about 0.5 fs. Characterization for the measurements done on sample A (see main text for designation) shows a pulse duration of 7.2 ± 0.5 fs.

B) d-scan

In-situ dispersion-scan traces were acquired by placing a 10-um thick BBO into the sample holder which is placed in the (vented) vacuum chamber that is used for the experiments, coupling the generated blue light into a low-noise spectrometer (OceanInsight QEPro), and

recording spectra at different insertions of a pair of fused silica wedges. Pulse retrieval was carried out with the same method as for the IFROG traces, by representing the spectral phase profile with a polynomial expansion $\phi(\omega) = \sum_{k=2}^{4} a_k(\omega - \omega_0)^k$, and fitting the coefficients a_k and the central frequency ω_0. (The spectral intensity profile was determined from an independent measurement.) The result of a typical measurement is summarized in figure S5. In-situ characterization for the measurements done on samples B and C (see main text for designation) show pulse durations of 10.8 ± 0.4 fs.

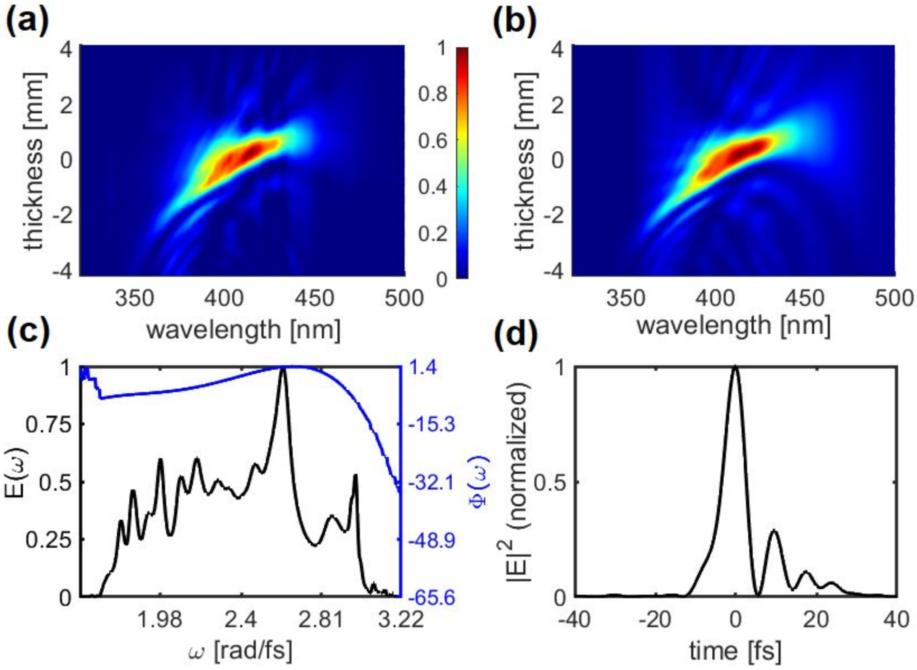

Figure S7. Typical d-scan measurement. **(a)** Experimental d-scan trace. **(b)** Retrieved trace. **(c)** Laser pulse spectrum (from independent measurement) and reconstructed spectral phase. **(d)** Reconstructed temporal intensity profile.

References

(1) Haynes, W. M.; Lide, D. R.; Bruno, T. J. *CRC Handbook of Chemistry and Physics*.

(2) Hohlfeld, J.; Wellershoff, S. S.; Güdde, J.; Conrad, U.; Jähnke, V.; Matthias, E. Electron and Lattice Dynamics Following Optical Excitation of Metals. *Chem. Phys.* **2000**, *251* (1–3), 237–258. https://doi.org/10.1016/S0301-0104(99)00330-4.

(3) Yurkevich, A. A.; Ashitkov, S. I.; Agranat, M. B. Permittivity of Gold with a Strongly Excited Electronic Subsystem. *Phys. Plasmas* **2017**, *24* (11), 164705. https://doi.org/10.1063/1.5000285.

(4) Hyyti, J.; Escoto, E.; Steinmeyer, G. Pulse Retrieval Algorithm for Interferometric Frequency-Resolved Optical Gating Based on Differential Evolution. *Rev. Sci. Instrum.* **2017**, *88* (10), 103102. https://doi.org/10.1063/1.4991852.
